# The duration-dependent and sex-specific effects of neonatal sevoflurane exposure on cognitive function in rats

**DOI:** 10.1590/1414-431X2024e13437

**Published:** 2024-05-20

**Authors:** Jiangxia Cheng, Zhuo Wang, Hui Yu, Ye Chen, Zhengchao Wang, Liangcheng Zhang, Xiaohong Peng

**Affiliations:** 1 Department of Anesthesia, Wuhan Fourth Hospital, Wuhan, China; 2 Department of Anesthesia, Fujian Medical University Union Hospital, Fuzhou, China; 3 Department of Orthopedics, Wuhan Fourth Hospital, Wuhan, China

**Keywords:** Sevoflurane, Cognitive function, Apoptosis, Synaptogenesis, Anesthesia

## Abstract

Clinical studies have found that neonatal sevoflurane exposure can increase the risk of cognitive dysfunction. However, recent studies have found that it can exhibit neuroprotective effects in some situations. In this study, we aimed to explore the effects of sevoflurane neonatal exposure in rats. A total of 144 rat pups (72 males and 72 females) were assigned to six groups and separately according to sevoflurane exposure of different times on the seventh day after birth. Blood gas analysis and western blot detection in the hippocampus were conducted after exposure. The Morris water maze test was conducted on the 32nd to 38th days after birth. The expression of PSD95 and synaptophysin in the hippocampus was detected after the Morris water maze test. We found that neonatal exposure to sevoflurane promoted apoptosis in the hippocampus, and Bax and caspase-3 were increased in a dose-dependent manner. The 2-h exposure had the greatest effects on cognitive dysfunction. However, with the extension of exposure time to 6 h, the effects on cognitive function were partly compensated. In addition, sevoflurane exposure decreased synaptogenesis in the hippocampus. However, as the exposure time was extended, the suppression of synaptogenesis was attenuated. In conclusion, neonatal sevoflurane exposure exhibited duration-dependent effects on cognitive function via Bax-caspase-3-dependent apoptosis and bidirectional effects on synaptogenesis in rats.

## Introduction

Sevoflurane is one of the most commonly used inhalational anesthetic drugs. This agent is useful for both inducing and maintaining anesthesia (1). In laboratory research, sevoflurane is commonly used to build animal models of cognitive dysfunction (2-4). The results of such studies have shown that sevoflurane can cause postoperative cognitive dysfunction via complex mechanisms, including neuroinflammation, changes in neurotransmitters, and a reduction in brain-derived neurotrophic factor (5). In recent years, studies have found that sevoflurane may exhibit neuroprotective effects in animal models, attenuating cognitive dysfunction via upregulation of SIRT1 and downregulation of caspase-1-mediated pyroptosis in the hippocampus in a sepsis mouse model anesthetized with tribromoethanol (6). Another study found that in cognitive impairments caused by hemorrhagic shock reperfusion, sevoflurane inhalation for 1 h at the onset of reperfusion could increase the expression of SIRT1 and bcl-2 and decrease the expression of Bax in the brain to attenuate cognitive dysfunction (7).

The adverse cognitive effects of sevoflurane seem to be most severe when exposure takes place in the neonatal period. In laboratory investigations, studies found that monkeys that were exposed to sevoflurane in infancy had heightened vulnerability to anxiety-related behaviors and anxious phenotypes during the process of growth (8). Mice exposed to sevoflurane in the neonatal period had an increased risk of cognitive dysfunction and attention-deficit/hyperactivity disorder-like impulsive behavior in later adulthood (9,10). However, clinical reports have been contradictory; one group reported that a single transient exposure to general anesthesia before 3 years of age had no relationship to poor neurodevelopmental outcomes (11,12), while others found it to be a risk factor (13). In general, clinical studies reported that exposure to sevoflurane in infancy and childhood could increase the risk of disorders of learning, motor function, and social ability (14,15). Thus, the effects of neonatal exposure to sevoflurane on the developmental process are still controversial and not fully clear.

In this study, we aimed to explore the duration-dependent and sex-specific effects of neonatal exposure to sevoflurane in mice, focusing on the effects and mechanisms related to cognitive function.

## Material and Methods

### Animals

All animal experimental protocols were reviewed and approved by the Animal Ethics Committee of Wuhan No. 4 Hospital, Wuhan, China. A total of 144 Sprague-Dawley rat pups (72 male and 72 female) were purchased from the Experimental Animal Center of Tongji Medical College, Huazhong University of Science and Technology (China). Nursing rat pups and their dams were housed one dam and litter per cage, with free access to food and water. The environment was controlled on a 12-h light/dark cycle (lights on at 08:00) at a temperature of 24°C. Nursing rat pups were raised by their dams until postnatal day 7 (P7, with P0 representing the date of birth), when they were randomized to six groups, and at least one animal from every litter was assigned to each of the six groups. There were 12 male rats and 12 female rats in each group. All efforts were made to minimize the number of rats used and their suffering. Male rats and female rats were detected separately in the following process.

### Neonatal exposure to sevoflurane

On P7, rats were randomly allocated to one of the following protocols for 3% sevoflurane exposure as was described in previous studies (16,17): continuous exposure for 0, 1, 2, 4, or 6 h or cumulative exposure for 6 h in 24 h (2 h sevoflurane followed by 6 h fresh air, repeated for 3 cycles). These groups were designated T1-T6 and T6in, respectively. All animals were kept in a 30% oxygen environment in an acrylic chamber within an incubator set to 37°C to maintain rectal temperatures of 36.5-37.5°C. The inhaled anesthetic and oxygen concentrations were controlled by an anesthesia apparatus (Avance CS^2^, General Electric, USA) and adjusted according to the instructions. Every 30 minutes, the pulse and peripheral oxygen saturation of the animals were measured by a handheld pulse oximeter (MD200K2, ChoiceMMed, USA). In each group, 6 male rats and 6 female rats were sacrificed by cervical dislocation for blood gas analysis and western blotting after the sevoflurane exposure period. The remaining rats were sacrificed in the same way for western blotting after the Morris water maze test.

### Blood gas analysis

At the end of exposure, left atrial puncture was performed at the second and third intercostal spaces of the nursing rat pups to obtain the arterial blood. A blood gas analysis (including pH, PaCO_2_, and PO_2_) was measured by a blood gas analyzer (GEM3500, USA).

### Western blots

The hippocampus of each rat was obtained for western blotting. The total protein content was extracted from the hippocampus, and protein concentrations were determined using a BCA protein assay kit (Pierce, ThermoFisher Scientific, USA). Total protein extract (40 g) was separated by 10% SDS-PAGE and then electrotransferred onto nitrocellulose membranes. After being blocked with TBST containing 5% skim milk, the membranes were incubated at 4°C overnight with primary antibodies (antibodies were diluted according to the manufacturer's instructions). The membranes were incubated with secondary antibodies at 37°C for 2 h (goat anti‐rabbit). After chromogen application, immunoreactive bands were obtained. Quantitative data were obtained from the results of densitometric methods by using AlphaEaseFC software (Alpha Innotec, Germany). Rabbit anti-cleaved caspase-3, Bax, PSD95, and synaptophysin GAPDH primary antibodies were purchased from Abcam (UK). Rabbit anti-glyceraldehyde 3-phosphate dehydrogenase (GAPDH) and horseradish peroxidase (HRP)-conjugated goat anti-rabbit IgG were obtained from Proteintech (USA).

### Morris water maze test

As summarized in a previously published review, the Morris water maze is a relevant tool to assess the mechanisms of both spatial learning and memory (18). Six male and 6 female rats in each group were returned to their dams. The Morris water maze (MWM) test was used to evaluate spatial learning and memory on P32-38 as described in previous studies (16,17). Briefly, the maze consisted of a round pool (painted black, 180 cm in diameter, 60 cm in height) filled with water heated to 22±2°C. For analysis, four equal quadrants, designated I, II, III, and IV, were defined within the pool. An escape platform (8 cm in diameter) was placed at the center of quadrant IV, approximately 1.5 cm below the water surface. Rats were subjected to four training sessions daily for five consecutive days to locate the hidden escape platform. Each trial started from a different quadrant and was limited to 90 s. If the rat reached the platform within 90 s, the time from beginning to end was considered the escape latency. If the rat failed to find the platform in the allotted time, the escape latency was recorded as 90 s, and the rat was placed onto the platform for 20 s. On day 6, a probe trial was performed by allowing the rat to swim for 90 s in the absence of the platform. The swimming time and trajectory of the rats were recorded by a Noldus EthoVision XT video analysis system (Noldus, Netherlands).

### Statistical analysis

Statistical analyses were performed using SPSS 21.0 software (IBM, USA). The normality of distribution was tested by a QQ plot. The data were analyzed using repeated-measures ANOVA. *Post hoc* pairwise tests were conducted, and the resulting P values were adjusted using the Bonferroni correction. An adjusted value of P<0.05 was considered to indicate statistical significance.

## Results

### Blood gas tests after sevoflurane exposure

After sevoflurane exposure, arterial blood was obtained for blood gas analysis. As shown in Table 1, the pH value decreased with increasing sevoflurane exposure time, while the PaCO_2_ increased, resulting in duration-dependent hypercapnia. The average partial pressure of CO_2_ in male rats was higher than that in female rats in the T1, T2, T4, and T6 groups, but these differences between sexes were not significant. The blood gas results were significantly different between the T6in and T6 groups, with the blood gas profile of the former group closely resembling that of the T2 group. As for the PO_2_, the T1, T2, and T4 groups had the highest PO_2_ levels, possibly because of the intervention of the anesthesia machine. As the exposure extended to 6 h, the PO_2_ decreased and became similar to that of the T0 group. The PO_2_ was significantly higher in the T6in group than in the T6 group, with the former resembling the T2 group.

**Table 1:** pH, partial pressure of CO_2_ (PCO_2_), and partial pressure of O_2_ (PO_2_) in arterial blood of rats after sevoflurane exposure (continuous exposure for 0, 1, 2, 4, or 6 h (T0 to T6) or cumulative exposure for 6 in 24 h (T6in)) separated by group and sex.

Group	T0	T1	T2	T4	T6	T6in
pH (male)	7.29±0.01	7.17±0.04	7.14±0.03*	7.17±0.04	7.00±0.14*^#&$^	7.16±0.10^!^
pH (female)	7.29±0.02	7.23±0.08*	7.20±0.44*	7.20±0.03*	7.02±0.15*^#&$^	7.21±0.04*^!^
PCO_2_ (male; mmHg)	63.50±3.83	80.00±13.87*	89.33±8.50*	80.80±5.81*	108.43±12.84*^#$^	83.00±5.76*^!^
PCO_2_ (female; mmHg)	61.00±2.83	73.50±15.13*	81.50±5.00*	76.20±8.70*	91.71±17.88*	81.50±5.01*
PO_2_ (male; mmHg)	28.50±1.52	43.50±9.57*	51.00±6.32*	49.60±9.91*	32.86±15.00^#&$^	54.00±2.37*^!^
PO_2_ (female; mmHg)	28.67±3.56	35.00±9.06*	54.17±2.64*^#^	55.20±5.93*^#^	30.43±14.07^&$^	54.17±2.64*^#!^

Data are reported as means and SD. *P<0.05/30 *vs* T0 group; ^#^P<0.05 *vs* T1 group; ^&^P<0.05 *vs* T2 group; ^$^P<0.05 *vs* T4 group; ^!^P<0.05 *vs* T6 group (ANOVA).

These results indicated that neonatal exposure to sevoflurane can lead to hypercapnia. Additionally, intermittent exposure could avert the hypercapnia caused by long exposure to sevoflurane.

### Sevoflurane promotes Bax-caspase-3-dependent apoptosis in the hippocampus

**Figure 1:**
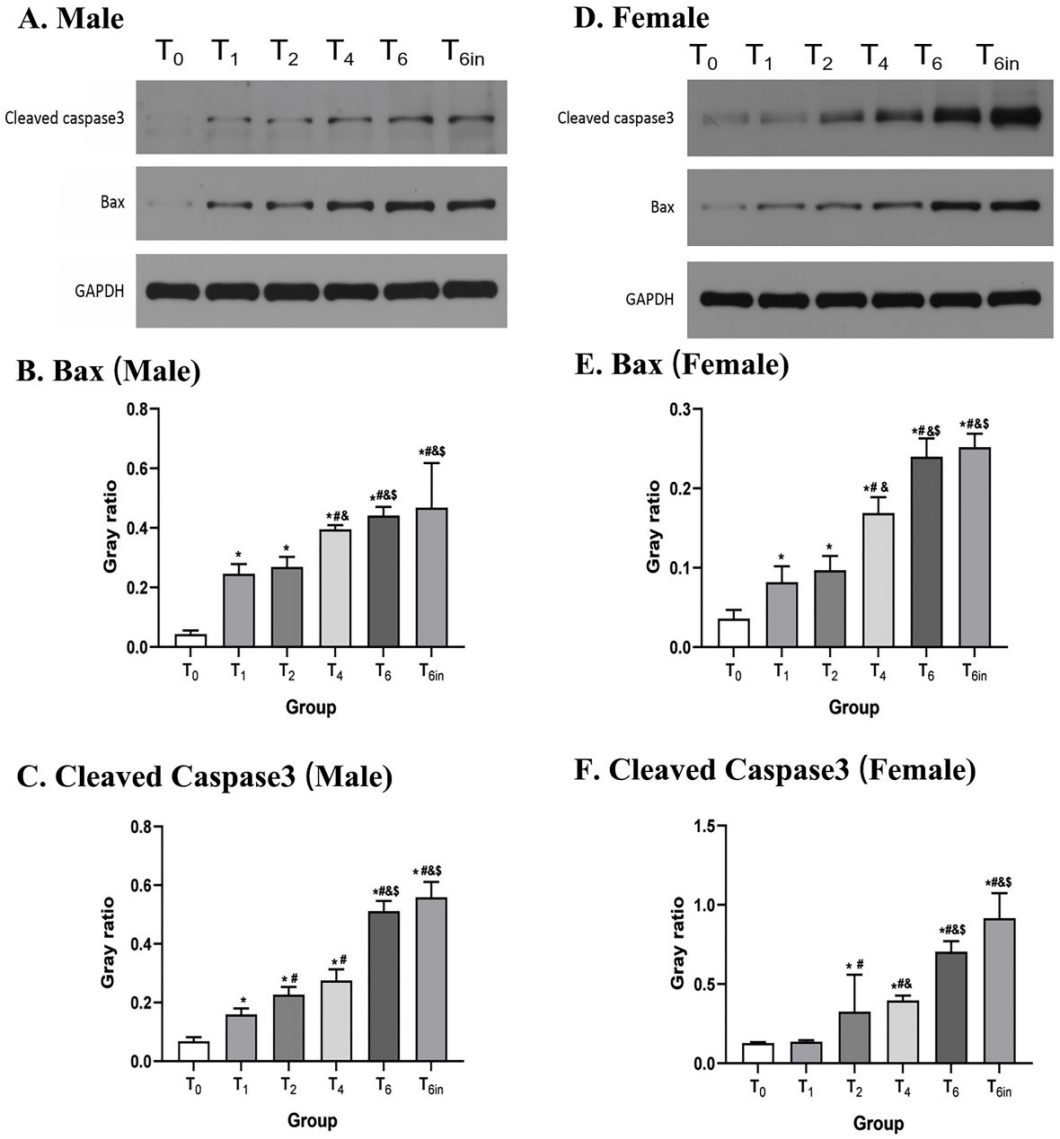
The levels of Bax and cleaved caspase-3 in the hippocampus after sevoflurane exposure (continuous exposure for 0, 1, 2, 4, or 6 h (T0 to T6) or cumulative exposure for 6 in 24 h (T6in)) were detected by western blot. **A**-**C**, The levels of Bax and cleaved caspase-3 in the hippocampus of male rats in each group. **D**-**F**, The levels of Bax and cleaved caspase-3 in the hippocampus of female rats in each group. The grouping of gels was cropped from different gels while the samples were derived from the same experiment. Data are reported as means and SD. *P<0.05 *vs* T0 group; ^#^P<0.05 *vs* T1 group; ^&^P<0.05 *vs* T2 group; ^$^P<0.05 *vs* T4 group (ANOVA).

The levels of Bax and cleaved caspase-3 in the hippocampus were measured after sevoflurane exposure. As shown in Figure 1, sevoflurane increased the expression of Bax and the activation of caspase-3 in both male and female rats. Specifically, with increasing sevoflurane exposure time, the levels of Bax and cleaved caspase 3 gradually increased. The results were not significantly different between the T6 and T6in groups. These results indicated that neonatal exposure to sevoflurane can promote apoptosis in the hippocampus via Bax and caspase-3.

### Bidirectional effect of sevoflurane on cognitive function

**Figure 2:**
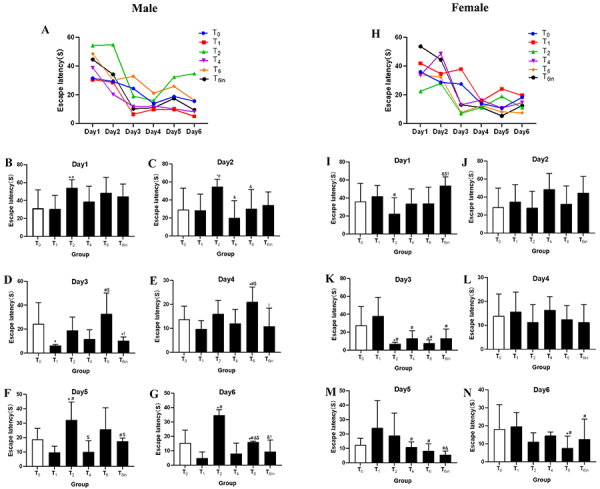
Escape latency obtained from the Morris water maze test after rats were placed in continuous exposure for 0, 1, 2, 4, or 6 h (T0 to T6) or cumulative exposure for 6 in 24 h (T6in). **A**, The overall results of 6-day-old male rats. **B**-**G**, The escape latency of male rats by group and day. **H**, The overall results of 6-day-old female rats. **I**-**N**, The escape latency of female rats by group and day. Data are reported as means and SD. *P<0.05 *vs* T0 group; ^#^P<0.05 *vs* T1 group; ^&^P<0.05 *vs* T2 group; ^$^P<0.05 *vs* T4 group; ^!^P<0.05 *vs* T6 group (ANOVA).

**Figure 3:**
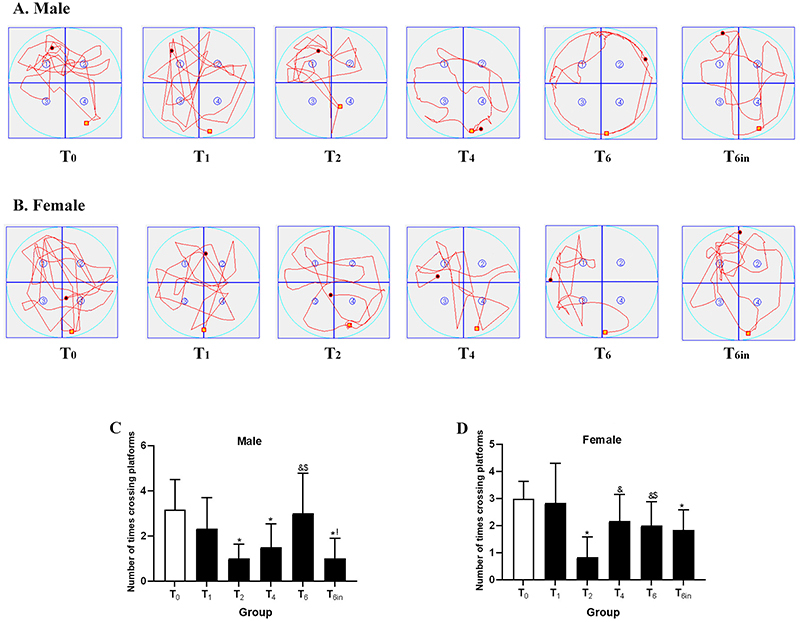
The number of platform crossings in the Morris water maze test after rats were placed in continuous exposure for 0, 1, 2, 4, or 6 h (T0 to T6) or cumulative exposure for 6 in 24 h (T6in). **A**, Latency graphs to reach the platform of male rats in each group. **B**, Latency graphs to reach the platform of female rats in each group. **C**, Number of platform crossings by male rats in each group. **D**, Number of platform crossings by female rats in each group. Data are reported as means and SD. *P<0.05 *vs* T0 group; ^&^P<0.05 *vs* T2 group; ^$^P<0.05 *vs* T4 group; ^!^P<0.05 *vs* T6 group (ANOVA).

The effects of neonatal exposure to sevoflurane on cognitive function were assessed using the Morris water maze test. As shown in Figure 2, among male rats, the escape latency was significantly longer in the T2 group than in the other groups. Meanwhile, the escape latency of the T6 group was the second longest. As shown in Figure 3, male rats in the T2 group had the lowest number of platform crossings among all males and had significantly fewer crossing than the T0 group. The male rats in the T4 and T6 groups also crossed the platform location a significantly greater number of times than those in the T2 group but showed no significant difference from the male rats in the T0 and T1 groups. In the female group, the results were quite different. As shown in Figure 2, the escape latency was not significantly different among the T0, T1, T2, and T4 groups. However, the escape latency of the T6 group was the shortest and was significantly shorter than those of the T0 and T1 group. Regarding the number of platform crossings, the T2 female group had the lowest number of crossings, with significantly fewer crossings than the T0 group (Figure 3). The number of platform crossings was also significantly lower in the T4 group than in the T0 group. However, there were no significant differences among the T0, T1, and T6 group. As for the T6in group, male rats in this group had a significantly shorter escape latency than those in the T6 group but still made fewer platform crossings than the T6 group. In female rats, the escape latency and the number of platform crossings were not significantly different between the T6 and T6in groups.

These results suggested that the effects of sevoflurane on cognitive function did not have a monotonic relationship with exposure time. The 2-h exposure had the greatest adverse effects on cognitive function. However, as the exposure time was extended to 6 h, the effects on cognitive function were diminished, which indicated a bidirectional effect of sevoflurane. Meanwhile, as evaluated by escape latency, female rats might have a better tolerance than male rats for neonatal sevoflurane exposure.

### Effects of sevoflurane on synaptogenesis in the hippocampus

**Figure 4:**
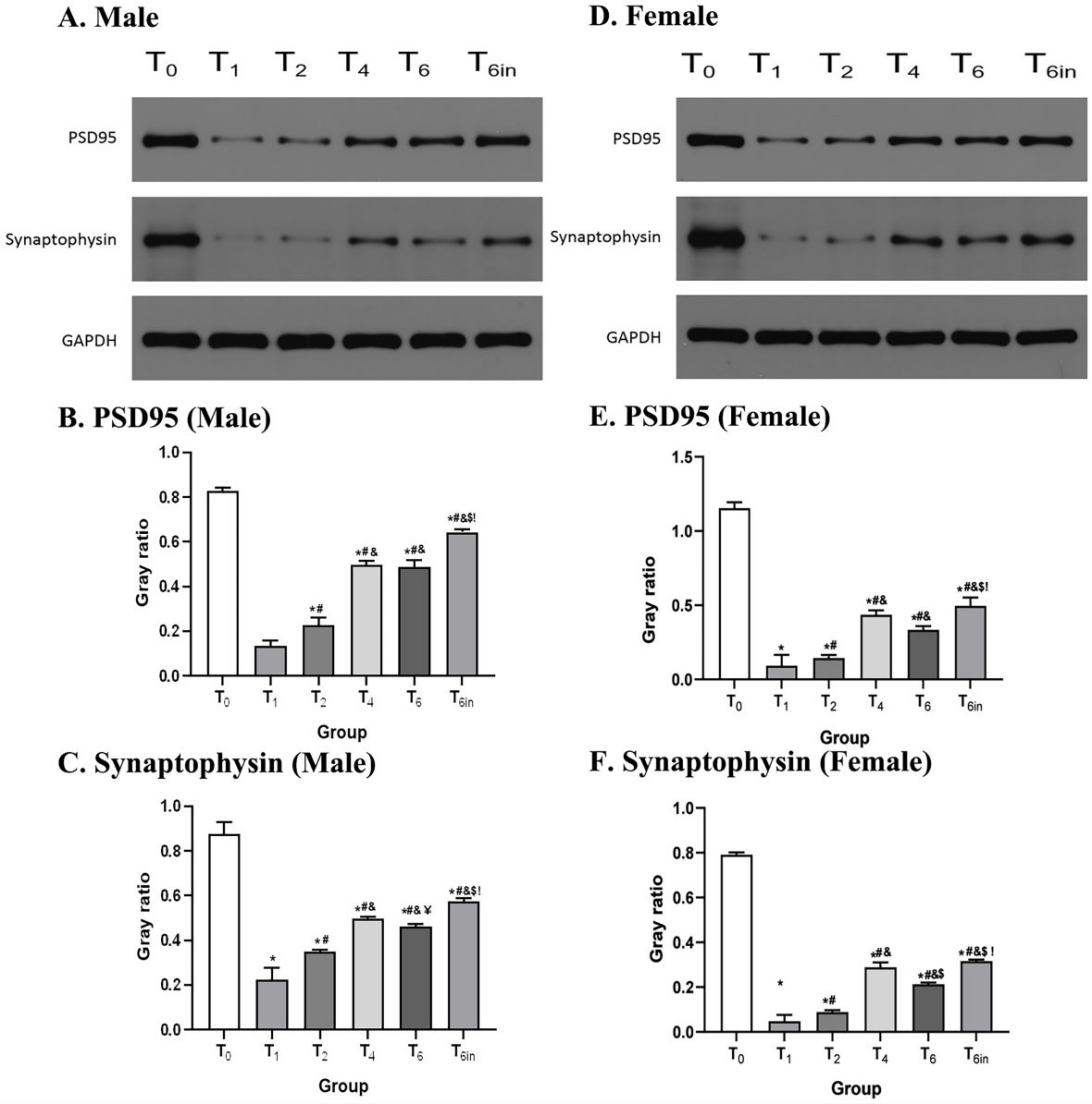
The levels of PSD95 and synaptophysin in the hippocampus after the Morris water maze test were detected by western blot after rats were placed in continuous exposure for 0, 1, 2, 4, or 6 h (T0 to T6) or cumulative exposure for 6 in 24 h (T6in). **A**-**C**, Levels of PSD95 and synaptophysin in the hippocampus of male rats in each group. **D**-**F**, Levels of PSD95 and synaptophysin in the hippocampus of female rats in each group. The grouping of gels was cropped from different gels while the samples were derived from the same experiment. Data are reported as means and SD. *P<0.05 *vs* T0 group; ^#^P<0.05 *vs* T1 group; ^&^P<0.05 *vs* T2 group; ^$^P<0.05 *vs* T4 group; ^!^P<0.05 *vs* T6 group (ANOVA).

After the Morris water maze test, the level of synaptogenesis in the hippocampus was evaluated by western blots for PSD95 and synaptophysin. As shown in Figure 4, among both males and females, the expression levels of PSD95 and synaptophysin were significantly decreased in the sevoflurane-exposed animals. Meanwhile, the expression levels of PSD95 and synaptophysin gradually increased with the extension of exposure time. The expression levels of PSD95 and synaptophysin in the T4 and T6 groups were significantly higher than those in the T1 and T2 groups. The results were not significantly different between the T6 and T6in groups. These results suggested that exposure to sevoflurane decreased synaptogenesis in the hippocampus. However, with the extension of exposure time, this reduction in synaptogenesis was attenuated, which could be one of the possible explanations for the bidirectional effect of sevoflurane on cognitive function.

## Discussion

In this study, we demonstrated that neonatal exposure to sevoflurane can lead to an increase in Bax-caspase-3-dependent apoptosis in the hippocampus. However, regarding the effects on cognitive function during subsequent growth and development, a relatively short exposure period of 2 h had the greatest effects. When the exposure time was extended to 6 h, the effects on cognitive function were diminished. Consistent with this result, synaptogenesis in the hippocampus was significantly decreased after a short exposure to sevoflurane, but the effects were gradually attenuated as the exposure time was extended to 6 h. Scholars have noted that hypoxia and hypercarbia caused by anesthesia can cause neurodevelopmental delay (19). Additionally, studies suggest that anesthesia by sevoflurane can lead to hypercapnia and produce a neurotoxic effect (20). Our results showed that the T6in group had significantly less hypercapnia than the T6 group, while the other measured results were basically similar, which implies that the latter effects were caused by sevoflurane, not sevoflurane-induced hypercapnia.

The effects of sevoflurane on apoptosis are quite different in different tissues and diseases. A study published in 2019 found that sevoflurane could exhibit antiapoptotic effects in lung tissue via NF-κB signaling in lipopolysaccharide (LPS)-induced pulmonary injury (21). However, another study published in 2018 found that sevoflurane could increase apoptosis in hepatocytes via a Bax/Bcl-2-dependent pathway to exhibit protective effects in ischemic liver injury (22). Other types of programmed cell death, including autophagy and pyroptosis, were also discovered to increase in nervous tissue due to the influence of sevoflurane (6,7). Nevertheless, the effects of this anesthetic on apoptosis in nervous tissue are still unclear. In this study, we measured the levels of Bax and cleaved caspase-3 in the hippocampus just after sevoflurane exposure at P7. Our results were basically consistent with a previous study (22) showing that sevoflurane could upregulate Bax-caspase-3-dependent apoptosis in the hippocampus. However, this result captured the effects of sevoflurane only in the short period after exposure. Whether this agent has other apoptosis-related effects on nervous tissue in the process of growth and development is still unclear and needs further study.

Regarding the effects on synaptogenesis, a previous study found that P6 mice treated with 3% sevoflurane 2 h daily for 3 consecutive days had significantly decreased synaptogenesis in the hippocampus (23). Furthermore, another study found that P7 rats receiving a single exposure to 2.5% sevoflurane for 6 h showed not only a decrease in synaptogenesis but also an increase in the proportion of symmetric synapses in the hippocampus at P30 (24). That study demonstrated that the effects of neonatal exposure to sevoflurane were not limited to decreased synaptogenesis in the process of growth (24); in particular, this agent could promote synaptogenesis to some extent, leading to a shift in the excitatory/inhibitory synapse balance (24). In the same study, cognitive function tested by the MWM on P23-25 in the 6 h sevoflurane-treated group was not much lower than that in the control group (24). In the results shown above, we found that cognitive function was most damaged in the 2 h sevoflurane-treated group, while the extension of sevoflurane exposure time reduced the adverse effect on cognitive function. The same results were obtained in the assessment of synaptogenesis-related proteins: short exposure led to a greater decrease in synaptogenesis, while long exposure led to a smaller decrease in synaptogenesis. Based on previous studies and our results, we suggested that sevoflurane would exhibit bidirectional effects on synaptogenesis depending on exposure time: the suppressive effects would predominate after a short exposure, whereas the promoting effects would gradually increase with exposure time. This time-dependent effect could partly avert the cognitive dysfunction caused by the neurotoxicity of sevoflurane. However, neonatal sevoflurane exposure has also been found to cause behavioral disorders, social disorders, and dysplasia in rodents, which might also be related to its unusual synaptogenesis-promoting effects (25-27).

The protective toxic effects that sevoflurane could exhibit depend on the condition of the brain. As outlined in the Introduction of this manuscript, despite sevoflurane's neurotoxicity, it could also exhibit neuroprotective effects in pyroptosis and hemorrhagic shock reperfusion (6,7). In this study, we discovered that the main differences between male and female rats were in the escape latencies during the MWM test, particularly in the T2 group. Male rats in the T2 group exhibited longer escape latencies compared to the T0 and T1 groups, while female rats in the T2 group had shorter escape latencies than the T0 and T1 groups. Based on previous studies, it is challenging to completely unveil the mechanisms behind this sex-dependent effect. Some studies have found that the differences in blood-brain barrier permeability may cause sevoflurane to exhibit distinct effects at different ages (28,29). As a result, we suspect that this phenomenon might be attributed to the slight differences in blood-brain barrier response when exposed to sevoflurane. However, the sex differences in neurodevelopmental abnormalities caused by anesthesia exposure and the underlying mechanism remain unclear and require further exploration (30).

This study had some limitations. First, although we clearly identified duration-dependent antiapoptotic effects in the early stage and bidirectional effects on synaptogenesis from the early stage to the late stage of sevoflurane exposure, the underlying mechanisms are still unclear and need to be further explored. Second, although we found some differences in the results of the blood gas analysis and MWM test between the different sexes, there is still insufficient evidence to draw a conclusion about which sex has better tolerance to neonatal sevoflurane exposure. Studies have raised concerns about the sex differences in neurodevelopmental abnormalities caused by neonatal anesthesia exposure (31). However, most related studies have used only one sex for research. The differential effects of sevoflurane on males and females are still unclear. We suspected that there could be some differences between sexes in the effects of sevoflurane involving multiple organs under the influence of hormones; this possibility needs to be further explored.

In conclusion, this study found that neonatal sevoflurane exposure exhibited duration-dependent effects on cognitive function in rats. Regarding the mechanisms, sevoflurane could lead to Bax-caspase-3-dependent apoptosis in the hippocampus at an early stage. In neurodevelopment, neonatal sevoflurane could exhibit time-dependent bidirectional effects on synaptogenesis, which might account for its bidirectional effects on cognitive function. The results of this study provided new insight into the application of sevoflurane in anesthesia, especially in pediatric anesthesia. Furthermore, the time-dependent results of this study could be referenced in further research using sevoflurane to induce cognitive dysfunction.

## References

[1] Sevoflurane (2012). LiverTox: Clinical and Research Information on Drug-Induced Liver Injury.

[2] Sun M, Dong Y, Li M, Zhang Y, Liang F, Zhang J (2021). Dexmedetomidine and clonidine attenuate sevoflurane-induced tau phosphorylation and cognitive impairment in young mice via α-2 adrenergic receptor. Anesth Analg.

[B03] Yang Y, Liang F, Gao J, Dong Y, Zhang Y, Yang G (2021). Testosterone attenuates sevoflurane-induced tau phosphorylation and cognitive impairment in neonatal male mice. Br J Anaesth.

[4] Chen Y, Zhang P, Lin X, Zhang H, Miao J, Zhou Y (2020). Mitophagy impairment is involved in sevoflurane-induced cognitive dysfunction in aged rats. Aging (Albany NY).

[5] Wang CM, Chen WC, Zhang Y, Lin S, He HF (2021). Update on the mechanism and treatment of sevoflurane-induced postoperative cognitive dysfunction. Front Aging Neurosci.

[6] Chen H, Peng Y, Wang L, Wang X (2022). Sevoflurane attenuates cognitive dysfunction and NLRP3-dependent caspase-1/11-GSDMD pathway-mediated pyroptosis in the hippocampus via upregulation of SIRT1 in a sepsis model. Arch Physiol Biochem.

[7] Shu J, Huang X, Liao Q, Wang J, Zhou Y, Chen Y (2022). Sevoflurane improves hemorrhagic shock and resuscitation-induced cognitive impairments and mitochondrial dysfunctions through SIRT1-mediated autophagy. Oxid Med Cell Longev.

[8] Raper J, Alvarado MC, Murphy KL, Baxter MG (2015). Multiple anesthetic exposure in infant monkeys alters emotional reactivity to an acute stressor. Anesthesiology.

[9] Dai CL, Li H, Hu X, Zhang J, Liu F, Iqbal K (2020). Neonatal exposure to anesthesia leads to cognitive deficits in old age: prevention with intranasal administration of insulin in mice. Neurotox Res.

[10] Xie L, Liu Y, Hu Y, Wang B, Zhu Z, Jiang Y (2020). Neonatal sevoflurane exposure induces impulsive behavioral deficit through disrupting excitatory neurons in the medial prefrontal cortex in mice. Transl Psychiatry.

[11] Davidson AJ, Disma N, de Graaff JC, Withington DE, Dorris L, Bell G (2016). Neurodevelopmental outcome at 2 years of age after general anaesthesia and awake-regional anaesthesia in infancy (GAS): an international multicentre, randomised controlled trial. Lancet.

[12] Sun LS, Li G, Miller TL, Salorio C, Byrne MW, Bellinger DC (2016). Association between a single general anesthesia exposure before age 36 months and neurocognitive outcomes in later childhood. JAMA.

[13] DiMaggio C, Sun LS, Li G (2011). Early childhood exposure to anesthesia and risk of developmental and behavioral disorders in a sibling birth cohort. Anesth Analg.

[14] Warner DO, Zaccariello MJ, Katusic SK, Schroeder DR, Hanson AC, Schulte PJ (2018). Neuropsychological and behavioral outcomes after exposure of young children to procedures requiring general anesthesia: the Mayo Anesthesia Safety in Kids (MASK) study. Anesthesiology.

[15] Walkden GJ, Gill H, Davies NM, Peters AE, Wright I, Pickering AE (2020). Early childhood general anesthesia and neurodevelopmental outcomes in the avon longitudinal study of parents and children birth cohort. Anesthesiology.

[16] Devroe S, Van der Veeken L, Bleeser T, Van der Merwe J, Meeusen R, Van de Velde M (2021). The effect of xenon on fetal neurodevelopment following maternal sevoflurane anesthesia and laparotomy in rabbits. Neurotoxicol Teratol.

[17] Yu Y, Zhang P, Yan J, Sun Y, Wu X, Xi S (2016). Sevoflurane induces cognitive impairments via the MiR-27b/LIMK1-signaling pathway in developing rats. Inhal Toxicol.

[18] Othman MZ, Hassan Z, Che Has AT (2022). Morris water maze: a versatile and pertinent tool for assessing spatial learning and memory. Exp Anim.

[19] Floyd TF, Khmara K, Lamm R, Seidman P (2020). Hypoxia, hypercarbia, and mortality reporting in studies of anaesthesia-related neonatal neurodevelopmental delay in rodent models: a systematic review. Eur J Anaesthesiol.

[20] Kung SC, Shen YC, Chang ET, Hong YL, Wang LY (2018). Hypercapnia impaired cognitive and memory functions in obese patients with obstructive sleep apnoea. Sci Rep.

[21] Wang Y, Zhang X, Tian J, Liu G, Li X, Shen D (2019). Sevoflurane alleviates LPS-induced acute lung injury via the microRNA-27a-3p/TLR4/MyD88/NF-κB signaling pathway. Int J Mol Med.

[22] Beck-Schimmer B, Z'graggen BR, Booy C, Köppel S, Spahn DR, Schläpfer M (2018). Sevoflurane protects hepatocytes from ischemic injury by reducing reactive oxygen species signaling of hepatic stellate cells: translational findings based on a clinical trial. Anesth Analg.

[23] Tao G, Luo Y, Xue Q, Li G, Tan Y, Xiao J (2016). Docosahexaenoic acid rescues synaptogenesis impairment and long-term memory deficits caused by postnatal multiple sevoflurane exposures. Biomed Res Int.

[24] Zhang W, Chen Y, Qin J, Lu J, Fan Y, Shi Z (2022). Prolonged sevoflurane exposure causes abnormal synapse development and dysregulates beta-neurexin and neuroligins in the hippocampus in neonatal rats. J Affect Disord.

[25] Zhong L, Ma X, Niu Y, Zhang L, Xue Z, Yan J (2022). Sevoflurane exposure may cause dysplasia of dendritic spines and result in fine motor dysfunction in developing mouse through the PI3K/AKT/mTOR pathway. Front Neurosci.

[B26] Useinovic N, Near M, Cabrera OH, Boscolo A, Milosevic A, Harvey R (2023). Neonatal sevoflurane exposure induces long-term changes in dendritic morphology in juvenile rats and mice. Exp Biol Med (Maywood).

[27] Zhou B, Chen L, Liao P, Huang L, Chen Z, Liao D (2019). Astroglial dysfunctions drive aberrant synaptogenesis and social behavioral deficits in mice with neonatal exposure to lengthy general anesthesia. PLoS Biol.

[28] Restin T, Kajdi ME, Schläpfer M, Z'graggen BR, Booy C, Dumrese C (2017). Sevoflurane protects rat brain endothelial barrier structure and function after hypoxia-reoxygenation injury. PloS One.

[29] Acharya NK, Goldwaser EL, Forsberg MM, Godsey GA, Johnson CA, Sarkar A (2015). Sevoflurane and Isoflurane induce structural changes in brain vascular endothelial cells and increase blood-brain barrier permeability: Possible link to postoperative delirium and cognitive decline. Brain Res.

[30] Aksenov DP, Miller MJ, Dixon CJ, Drobyshevsky A (2020). Impact of anesthesia exposure in early development on learning and sensory functions. Dev Psychobiol.

[31] Cabrera OH, Gulvezan T, Symmes B, Quillinan N, Jevtovic-Todorovic V (2020). Sex differences in neurodevelopmental abnormalities caused by early-life anaesthesia exposure: a narrative review. Br J Anaesth.

